# Identification and dual-center histological validation of EMT core genes in chronic rhinosinusitis with nasal polyps: an integrated multi-cohort transcriptomic and single-cell analysis

**DOI:** 10.3389/fimmu.2026.1774236

**Published:** 2026-03-17

**Authors:** Kai Xu, Yingjie Song, Mujun Shen, Jiahao Li, Kaiqi Chen, Hongyan Lai, Linglong Li, Feng Zhang, Lei Shi, Dehong Mao, Fu Shu

**Affiliations:** 1 College of Pharmaceutical Sciences, Southwest University, Chongqing, China; 2 Department of Otorhinolaryngology, Jiangsu Province Hospital of Chinese Medicine Chongqing Hospital (Chongqing Yongchuan Hospital of Chinese Medicine), Chongqing, China; 3 Department of Otorhinolaryngology, The Affiliated Hospital of Liaoning University of Traditional Chinese Medicine, Shenyang, China; 4 Yibin Academy of Southwest University, Yibin, China

**Keywords:** bioinformatics, chronic rhinosinusitis with nasal polyps, diagnostic biomarker, epithelial-mesenchymal transition, machine learning

## Abstract

**Background:**

Chronic rhinosinusitis with nasal polyps (CRSwNP) is highly heterogeneous. Epithelial–mesenchymal transition (EMT) is implicated in mucosal remodeling and postoperative recurrence, yet robust EMT biomarkers consistently validated across cohorts and by histology are lacking.

**Methods:**

RNA-seq data from a Chongqing (CQ) cohort were integrated with multiple GEO datasets. After batch-effect correction, differential expression analysis and weighted gene co-expression network analysis (WGCNA) were performed. EMT-related candidates were obtained by intersecting results with the MSigDB EMT gene set. Core genes were identified using multi-algorithm feature selection (LASSO, SVM-RFE, and random forest). A three-gene model was constructed and externally validated. Single-cell transcriptomic data were used to define cellular sources of core genes, and immune infiltration and pathway activity were assessed. Regulatory networks (TF/miRNA) and compound–disease associations were predicted. Finally, expression was validated in dual-center clinical cohorts (CQ and Liaoning [LN]) by qRT-PCR and immunohistochemistry/immunofluorescence, and associations with SNOT-22 and the eosinophilic endotype were evaluated.

**Results:**

Twenty-five EMT-related candidate genes were identified. Multi-algorithm intersection highlighted SPP1, PTHLH, and IGFBP3 as EMT core genes, consistently upregulated in the training set, the external validation dataset, and dual-center specimens. The three-gene model achieved AUCs of 0.944–0.991 in the training set and 0.888–0.938 in the external validation dataset. Single-cell mapping indicated that SPP1 was primarily derived from myeloid cells, PTHLH from epithelial cells, and IGFBP3 enriched in fibroblasts. Higher core-gene expression was associated with increased immune infiltration and activation of TGF-β, hypoxia/glycolysis, and inflammation-related pathways. Histology supported EMT-associated phenotypic changes in CRSwNP, with stronger signals in the eosinophilic endotype. In CQ and LN cohorts, core-gene expression correlated with SNOT-22 (Spearman r = 0.402–0.569, P ≤ 0.021).

**Conclusions:**

SPP1, PTHLH, and IGFBP3 are robustly validated EMT core genes in CRSwNP across multiple cohorts and dual-center histology, closely linked to immune microenvironment alterations and mucosal remodeling. These genes represent robust EMT-associated candidate biomarkers for future stratification efforts and mechanistic investigations.

## 1 Introduction

Chronic rhinosinusitis (CRS) is a chronic inflammatory disorder involving the mucosa of the nasal cavity and paranasal sinuses (1). Epidemiological studies indicate that CRS affects approximately 5%-12% of the general population and leads to substantial impairment in quality of life, increased healthcare utilization, and productivity loss (1–3). Clinically, CRS is commonly classified into CRS without nasal polyps (CRSsNP) and CRS with nasal polyps (CRSwNP) based on the presence of nasal polyps (1, 2). Compared with CRSsNP, CRSwNP is more likely to present with persistent nasal obstruction and olfactory dysfunction and has a more pronounced tendency toward refractoriness and recurrence. Comorbid lower airway diseases such as asthma are also more common and are associated with a higher symptom burden and more complex long-term management needs (4, 5). Current standard treatment for CRSwNP is based on saline irrigation and intranasal corticosteroids, with short courses of systemic corticosteroids and endoscopic sinus surgery (ESS) when indicated (6, 7). However, even with guideline-concordant medical and surgical interventions, a considerable proportion of patients experience suboptimal symptom control or postoperative recurrence during follow-up, and some patients require revision surgery (7, 8). In recent years, biologics have shown favorable efficacy and safety in CRSwNP, yet limited accessibility and the financial burden remain major barriers to widespread use (9–11). Therefore, beyond the traditional ‘anti-inflammatory plus surgery’ paradigm, identifying key pathological processes and molecular targets that are more accessible and readily translatable is of practical importance for optimizing long-term management strategies (12).

Beyond inflammatory cell infiltration, accumulating evidence suggests that epithelial-mesenchymal transition (EMT) is one of the key pathological underpinnings of CRSwNP development and mucosal remodeling (13–15). During EMT, epithelial cells lose polarity and intercellular adhesion, undergo cytoskeletal reorganization, and acquire mesenchymal-like phenotypes (16). In CRSwNP tissues, previous studies have observed downregulation of the epithelial marker E-cadherin and upregulation of mesenchymal-related molecules such as TGF-β1, α-SMA, fibronectin, and vimentin (17, 18). CRSwNP is often accompanied by prominent eosinophilic infiltration and persistently high expression of type 2 cytokines such as IL-4. Excessive inflammatory responses not only compromise epithelial barrier function but may also induce EMT through multiple inflammation-related signaling pathways. More importantly, sustained activation of EMT may contribute to remodeling of mucosal architecture and the extracellular matrix, leading to structural changes that are difficult to reverse, thereby maintaining a chronic inflammatory microenvironment and increasing treatment difficulty (13, 19). Interventions targeting EMT and the associated mucosal remodeling process have been proposed as potential entry points to alleviate CRSwNP progression (14, 18). Nevertheless, the key molecular networks and driving mechanisms of EMT in CRS have not been fully elucidated, and effective therapeutic options targeting the EMT-remodeling axis remain limited. In addition, there remains a need for EMT-associated core molecular features that can be stably reproduced across independent cohorts/platforms and supported by histological validation, to prioritize candidates for downstream mechanistic interrogation and prospective clinical evaluation.

Given the marked heterogeneity of CRSwNP and the complex interplay among inflammation, barrier dysfunction, and remodeling, studies based on a single omics layer or a single cohort often fail to consistently capture reproducible EMT-associated molecular features (20). Multi-cohort integration and cross-platform validation can improve signal robustness and reproducibility and can also localize the source cells and potential functional pathways of key genes at cellular resolution (21). In this study, we integrated multi-cohort bulk transcriptomic data, machine learning–based feature selection, and single-cell mapping to identify a robust EMT-associated core molecular signature and delineate its predominant cellular sources in CRSwNP. Bulk RNA-seq for the CQ cohort was performed early during cohort accrual, according to a prespecified plan, on the first consecutively enrolled CQ specimens that met predefined RNA quality thresholds (n = 12; 7 CRSwNP and 5 nasal septal deviation controls). This locally generated CQ RNA-seq subset was then integrated with multiple public datasets to construct the training set (**Supplementary Table S3**). Core findings were reproduced in an independent external GEO cohort and validated in dual-center clinical cohorts (CQ and Liaoning [LN]) that did not overlap with the training set. Histological and transcriptional validation (qRT-PCR, IHC, and IF) was performed across normal controls, eosinophilic CRSwNP, and non-eosinophilic CRSwNP tissues to support the robustness of the candidate biomarkers. Importantly, this study is exploratory and cross-sectional in nature; it provides candidate biomarkers and testable hypotheses but does not establish causal mechanisms or deliver clinically actionable stratification algorithms.

## 2 Methods

### 2.1 Study design and overall workflow

**Figure 1:**
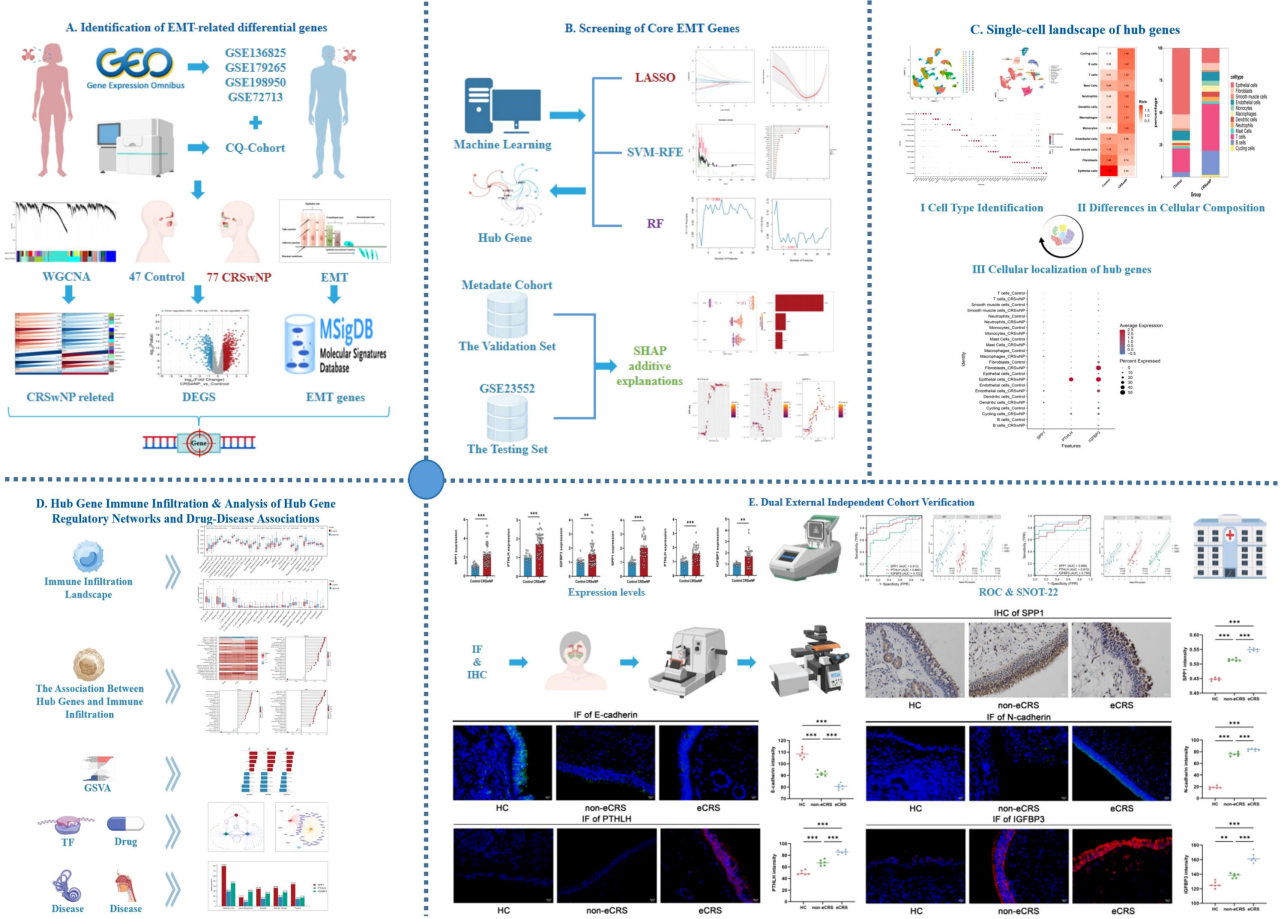
Study design and analytical workflow for identifying EMT core genes in CRSwNP. (A) Identification of EMT-related differentially expressed genes by integrating GEO datasets and the CQ cohort using WGCNA, differential expression analysis, and MSigDB-derived EMT gene sets. (B) Screening of core EMT genes using machine-learning algorithms (LASSO, SVM-RFE, and RF) and SHAP-based model interpretation. (C) Single-cell landscape of hub genes, including cell type identification, differences in cellular composition, and cellular localization of hub genes. (D) Immune infiltration analysis, regulatory network analysis, and drug-disease association analysis of hub genes. (E) Validation in two external independent cohorts by expression analysis, ROC and SNOT-22 analyses, and IHC/IF staining. EMT, epithelial–mesenchymal transition; CRSwNP, chronic rhinosinusitis with nasal polyps; GEO, Gene Expression Omnibus; WGCNA, weighted gene co-expression network analysis; MSigDB, Molecular Signatures Database; LASSO, least absolute shrinkage and selection operator; SVM-RFE, support vector machine–recursive feature elimination; SHAP, Shapley additive explanations; GSVA, gene set variation analysis; TF, transcription factor; miRNA, microRNA; qRT-PCR, quantitative real-time PCR; IHC, immunohistochemistry; IF, immunofluorescence; ROC, receiver operating characteristic; SNOT-22, 22-item Sino-Nasal Outcome Test; CQ, Chongqing cohort; LN, Liaoning cohort; HC, healthy control; eCRS, eosinophilic chronic rhinosinusitis.

We first integrated bulk transcriptomic data from the CQ cohort and four public datasets to construct the training set, followed by identification of EMT candidate genes, selection of a parsimonious core-gene signature, and development of a combinational mode. Expression replication and clinical correlation validation were subsequently performed in an independent external public dataset not included in the training set, as well as in dual-center clinical cohorts. Single-cell transcriptomic data were used to characterize the cellular origins of the core genes at cell-type resolution, and immune infiltration and pathway activity were further evaluated. In addition, transcription factor (TF) associations and potential intervention compounds and disease associations were explored in a hypothesis-generating manner. Finally, qRT-PCR and IHC/IF were performed for histological (**Figure 1**; **Supplementary Tables S1**–**S3**).

### 2.2 Collection and endotyping of clinical tissue samples

This study included clinical cohorts from two independent centers: the CQ cohort (Yongchuan Hospital of Traditional Chinese Medicine, Chongqing Medical University) and the LN cohort (Affiliated Hospital of Liaoning University of Traditional Chinese Medicine). According to the European Position Paper on Rhinosinusitis and Nasal Polyps (EPOS) criteria (1), baseline clinical data were collected from patients with CRSwNP in both cohorts and nasal polyp tissues were obtained; control tissues were collected from the uncinate process or middle turbinate of patients with nasal septal deviation without a clinical diagnosis of CRS and without endoscopic/radiologic evidence of CRS. To reduce confounding, patients with acute infection, fungal rhinosinusitis, or cystic fibrosis were excluded, as were those who had used corticosteroids, immunomodulators, or antibiotics within the previous month. In addition, patients with major comorbid conditions that could substantially influence EMT-related gene expression (e.g., systemic inflammatory/autoimmune disease, clinically significant fibrotic disease, or neoplastic disease) were excluded based on medical history and preoperative evaluation. To minimize treatment-related confounding, we restricted enrollment to biologic-naïve and surgery-naïve CRSwNP patients; individuals with prior exposure to biologic therapy or a history of sinonasal surgery (revision cases) were excluded, and thus all included CRSwNP patients were primary cases. Ultimately, the CQ cohort included 17 cases of nasal septal deviation and 40 cases of CRSwNP, and the LN cohort included 14 cases of nasal septal deviation and 29 cases of CRSwNP. In the LN cohort, samples were endotyped based on tissue eosinophil infiltration: eosinophilic CRSwNP (ECRSwNP) was defined as eosinophils ≥ 10 cells/high-power field (HPF), and the remaining cases were classified as non-eosinophilic CRSwNP (NECRSwNP) (1, 13). Clinical characteristics and sample information for both cohorts, including assay-specific sample allocation across RNA-seq and downstream validation experiments, are summarized in **Supplementary Tables S1**, **S2**.

### 2.3 Acquisition of internal transcriptomic sequencing data from the CQ cohort

Nasal tissue samples from the CQ cohort were selected for RNA sequencing (RNA-seq) according to procedures described in our previous studies (13, 21). In brief, freshly collected tissues were snap-frozen in liquid nitrogen and stored at −80 °C until processing. The control group included 5 patients with nasal septal deviation and the CRSwNP group included 7 patients. Total RNA was extracted using TRIzol. RNA concentration and purity were evaluated by NanoDrop 400A, and RNA integrity was assessed using an Agilent Bioanalyzer 2100. Samples meeting predefined quality control thresholds (RNA concentration > 50 ng/μL, total RNA > 1 μg, A260/280 > 1.8, and RIN > 7.0) were used for downstream library preparation. mRNA sequencing libraries were constructed according to the manufacturer’s protocol and paired-end sequencing was performed by BioMarker Co., Ltd. on an Illumina NovaSeq 6000 platform. Gene expression levels were quantified as fragments per kilobase of transcript per million mapped reads (FPKM).

### 2.4 Construction of the training set and definition of the external validation set

CRSwNP-related transcriptomic datasets GSE72713 (22), GSE136825 (23), GSE179265 (24), and GSE198950 (25) were downloaded from the GEO database and merged with the CQ cohort RNA-seq data after unifying gene identifiers to generate the training expression matrix. After preprocessing and harmonization, the final integrated training set consisted of 77 CRSwNP and 47 control samples (**Supplementary Table S3**). Batch effects were corrected using the ComBat method, and the correction effect was assessed by principal component analysis (PCA) (21, 26). The batch-corrected expression matrix was used for subsequent differential expression analysis, co-expression network construction, and machine learning-based screening. GSE23552 was additionally downloaded as an external validation dataset to validate differential expression directionality of the core genes and the discriminative performance of the model in an independent cohort (27).

### 2.5 Differentially expressed gene analysis

Differential expression analysis between CRSwNP and control samples in the training set was performed using the R package limma (version 3.40.6) (28). The thresholds for differentially expressed genes (DEGs) were set as P < 0.05 and |log2FC| > 0.5 (29).

### 2.6 Weighted gene co-expression network analysis

Based on the batch-corrected expression matrix, a weighted gene co-expression network was constructed using the R package WGCNA to identify co-expression modules significantly associated with the CRSwNP phenotype (30). Gene variability was first calculated using the median absolute deviation (MAD), and the top 25% most variable genes were retained for network construction. Samples were clustered to detect and remove potential outliers. An appropriate soft-thresholding power was then selected to build the co-expression network and generate the topological overlap matrix (TOM), and hierarchical clustering based on TOM dissimilarity was used to define modules. Module eigengenes were correlated with phenotype, with particular focus on modules showing the strongest positive and strongest negative correlations with CRSwNP status. Genes within these key modules were retained as candidates for subsequent EMT-related candidate gene screening and in-depth analyses (31).

### 2.7 Screening and functional annotation of EMT-related candidate genes

DEGs, genes from key WGCNA modules, and the EMT gene set in the MSigDB Hallmark collection were intersected to obtain the EMT candidate gene set (32). Spearman correlation analysis was used to evaluate correlations among candidate genes. Functional enrichment was performed using GO and KEGG analyses (clusterProfiler). Chromosomal locations of candidate genes were organized and visualized using Circos based on gene annotation information. Subcellular localization information was retrieved from the Human Protein Atlas (HPA) database.

### 2.8 Machine learning-based identification of EMT core genes and model construction

Multiple feature selection algorithms were applied to improve robustness, including LASSO regression (33), support vector machine recursive feature elimination (SVM-RFE) (34), and random forest (35). Core genes were defined as the intersection of genes selected by these algorithms. Diagnostic models were then constructed using multiple classifiers (e.g., LDA, Ridge, GBM, XGBoost, naive Bayes), and discriminative performance was evaluated by the area under the receiver operating characteristic curve (AUC) in both the training and external validation datasets. Model interpretability was assessed using SHAP to quantify feature contributions (36).

### 2.9 Immune infiltration and pathway activity analyses

Immune infiltration was assessed using two complementary approaches, ssGSEA and CIBERSORT (37, 38). ssGSEA was used to calculate relative enrichment scores of predefined immune cell gene sets in each sample, whereas CIBERSORT was used to estimate the relative proportions of 22 immune cell types. Spearman correlation analysis was performed to evaluate associations between EMT core genes and immune cell infiltration. Pathway activity was analyzed using Gene Set Variation Analysis (GSVA). Samples were divided into high- and low-expression groups according to the median expression of the core genes, and limma was used to compare GSVA scores between groups. The screening thresholds were |t| > 2 and P < 0.05, and results were visualized using t values/difference scores (21, 39).

### 2.10 TF regulatory network prediction and compound/disease association analysis

Candidate TF regulating SPP1, PTHLH, and IGFBP3 were predicted using the ChEA3 database via the miRNet platform, and a TF-gene network was constructed (40–42). To explore potentially translatable intervention clues, chemical interaction information for the core genes was retrieved from the Comparative Toxicogenomics Database (CTD). Compound-gene relationships supported by at least three publications were selected for network visualization (43). In addition, CTD-derived associations between the core genes and otorhinolaryngology-related diseases were extracted and visualized for comparison (44).

### 2.11 Single-cell transcriptomic analysis and cellular source localization

Single-cell transcriptomic data from 5 normal controls and 11 CRSwNP cases in the HRA000772 dataset were processed and analyzed using Seurat (45). Quality control retained cells with nFeature_RNA > 500 and removed cells with percent.mt > 25%. After normalization (NormalizeData), the top 2,000 highly variable genes were identified using FindVariableFeatures (vst method), followed by scaling with ScaleData. Harmony was used for integration and batch correction across samples/batches. Clustering was performed by constructing a neighbor graph based on PCA, and UMAP was used for dimensionality reduction and visualization. Cluster marker genes were identified using FindAllMarkers (thresholds: |log2FC| > 0.26, P < 0.01), and cell types were annotated based on canonical markers and our previous work (21, 46, 47). We focused on cell-type composition and cell-type-resolved expression of the EMT core genes.

### 2.12 Dual-center qRT-PCR validation and clinical correlation analysis

Total RNA was extracted from nasal tissues in the CQ and LN clinical cohorts, reverse-transcribed into cDNA, and the expression of the core genes was quantified by qRT-PCR. After normalization to internal reference genes, relative expression was calculated using the 2^-ΔΔCt^ method. Expression differences between CRSwNP and control groups were compared, and ROC curves were generated based on expression values to calculate AUCs. Spearman correlation analysis was used to evaluate associations between core-gene expression and SNOT-22 scores. The specific specimens included in qRT-PCR and correlation analyses are indicated in **Supplementary Tables S1**, **S2**. Primer sequences and qRT-PCR conditions are provided in **Supplementary Table S3**.

### 2.13 Histological validation

Histological validation of the selected core genes was performed in tissues from healthy controls (HC), NECRSwNP, and ECRSwNP. Detailed sample-level information for the histological validation subset (including endotyping and cohort source) is provided in **Supplementary Tables S1**, **S2**. The canonical EMT markers E-cadherin and N-cadherin were detected by IF. Among the core genes, SPP1 was examined by IHC, whereas PTHLH and IGFBP3 were examined by IF. IF and IHC staining procedures followed our previously published protocols with optimization for the antibodies used in this study (13, 48). Briefly, paraffin sections were deparaffinized and rehydrated, followed by antigen retrieval and blocking. Primary antibodies were incubated overnight at 4 °C. For IF, corresponding fluorescent secondary antibodies were applied and nuclei were counterstained with DAPI. For IHC, HRP-labeled secondary antibodies and DAB development were used, followed by counterstaining. Images were acquired under consistent microscopy and imaging settings. Quantitative image analysis was performed using a unified pipeline: IF signal levels were represented by mean fluorescence intensity of the target channel, and IHC protein levels were represented by the intensity of DAB brown staining. These metrics were compared among HC, NECRSwNP, and ECRSwNP groups. Antibody sources, catalog numbers, and dilution ratios are provided in **Supplementary Table S4**.

### 2.14 Statistical analysis

Statistical analyses were performed using R (v4.4.1) and GraphPad Prism (v9.5.1). Normally distributed data are presented as mean ± standard deviation and were analyzed using the independent-samples t test. Non-normally distributed data are presented as median (IQR) and were analyzed using the Mann-Whitney U test. Comparisons among three or more groups were performed using one-way ANOVA (Tukey *post hoc* test) or the Kruskal-Wallis test (Dunn multiple-comparison correction). Categorical variables were analyzed using the chi-square test or Fisher’s exact test. Correlations were assessed using Spearman’s method. ROC curves were used to evaluate diagnostic performance and calculate AUC. A two-sided P < 0.05 was considered statistically significant.

## 3 Results

### 3.1 Integration of multi-cohort training set, differential expression, and identification of EMT candidate genes

**Figure 2:**
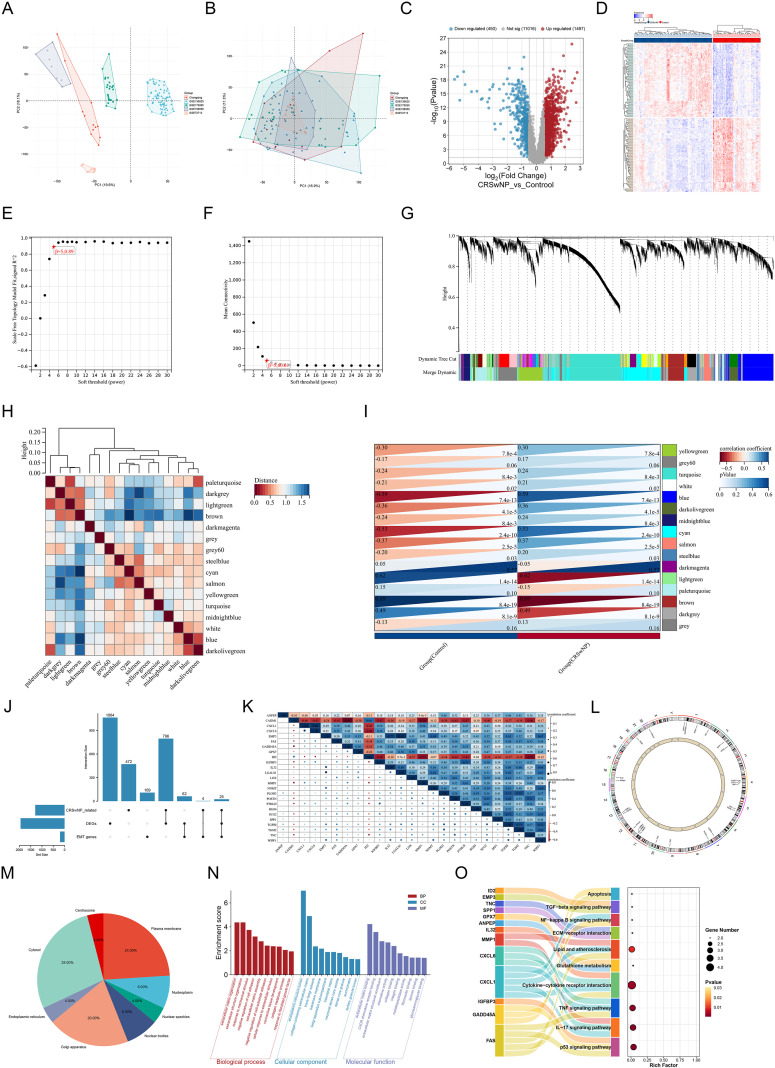
Multi-cohort integration identifies EMT-related candidate genes in CRSwNP. **(A, B)** Principal component analysis (PCA) before **(A)** and after **(B)** ComBat-based batch-effect correction across the integrated CQ cohort and GEO datasets. **(C)** Volcano plot of DEGs between CRSwNP and controls in the training set. **(D)** Heatmap of the top 100 differentially expressed genes across samples. **(E, F)** Determination of the WGCNA soft-thresholding power using the scale-free topology fit index **(E)** and mean connectivity **(F)**. **(G)** Gene dendrogram with module assignments (colored modules). **(H)** Clustering of module eigengenes illustrating inter-module relationships. **(I)** Module–trait associations (control vs CRSwNP), with correlation coefficients and P values. **(J)** UpSet plot showing the overlap among DEGs, CRSwNP-associated module genes, and the MSigDB Hallmark EMT gene set. **(K)** Spearman correlation matrix of the 25 candidates. **(L)** Circos plot depicting chromosomal distribution of candidate genes. **(M)** Subcellular localization of candidate-gene products based on the Human Protein Atlas. **(N)** GO enrichment analysis across BP, CC, and MF categories. **(O)** KEGG pathway enrichment analysis of EMT-related candidate genes. PCA, principal component analysis; DEGs, differentially expressed genes; WGCNA, weighted gene co-expression network analysis; MSigDB, Molecular Signatures Database; EMT, epithelial–mesenchymal transition; GO, Gene Ontology; BP, biological process; CC, cellular component; MF, molecular function; KEGG, Kyoto Encyclopedia of Genes and Genomes; CQ, Chongqing cohort.

After integrating CQ cohort RNA-seq data with four GEO datasets and correcting batch effects using ComBat, PCA showed markedly reduced separation of samples from different data sources (**Figures 2A, B**). The final training expression profile consisted of 77 patients with CRSwNP and 47 control samples. Differential expression analysis identified 1,947 DEGs, including 1,497 upregulated and 450 downregulated genes (**Figure 2C**), and a clustered heatmap of the top 100 DEGs is shown in **Figure 2D**. A WGCNA network was constructed based on the batch-corrected expression matrix; with a soft-thresholding power β = 5, the network satisfied scale-free topology criteria (**Figures 2E, F**). Module-phenotype correlation analysis showed that the modules most significantly positively and negatively correlated with CRSwNP status reached r = 0.59 (P = 7.4 × 10^-13^) and r = -0.69 (P = 8.4 × 10^-19^), respectively (**Figures 2G–I**). By intersecting DEGs, key module genes, and the EMT gene set, 25 EMT candidate genes were obtained (**Figure 2J**). Spearman correlation analysis showed an overall positive correlation trend among these candidate genes (**Figure 2K**).

### 3.2 Molecular characteristics and functional enrichment of EMT candidate genes

Circos analysis showed that the 25 EMT candidate genes were distributed across multiple autosomes, with no specific aggregation in a single chromosomal region (**Figure 2L**). According to HPA-based subcellular localization, the encoded proteins were mainly localized to the cytoplasm (28%), plasma membrane (24%), and Golgi apparatus (20%), with the remainder distributed in nucleoplasm/nuclear structures (20% in total) and a small proportion in the endoplasmic reticulum, centrosome, and other compartments (**Figure 2M**). GO enrichment indicated that the candidate genes were significantly involved in processes such as extracellular matrix organization, extracellular structure remodeling, inflammation/bacteria-related responses, and cytokine-mediated signaling (**Figure 2N**). KEGG analysis showed close associations with inflammation-remodeling-related pathways, including ECM-receptor interaction, TGF-β, TNF, IL-17, and NF-κB (**Figure 2O**).

### 3.3 Identification of EMT core genes using machine learning

**Figure 3:**
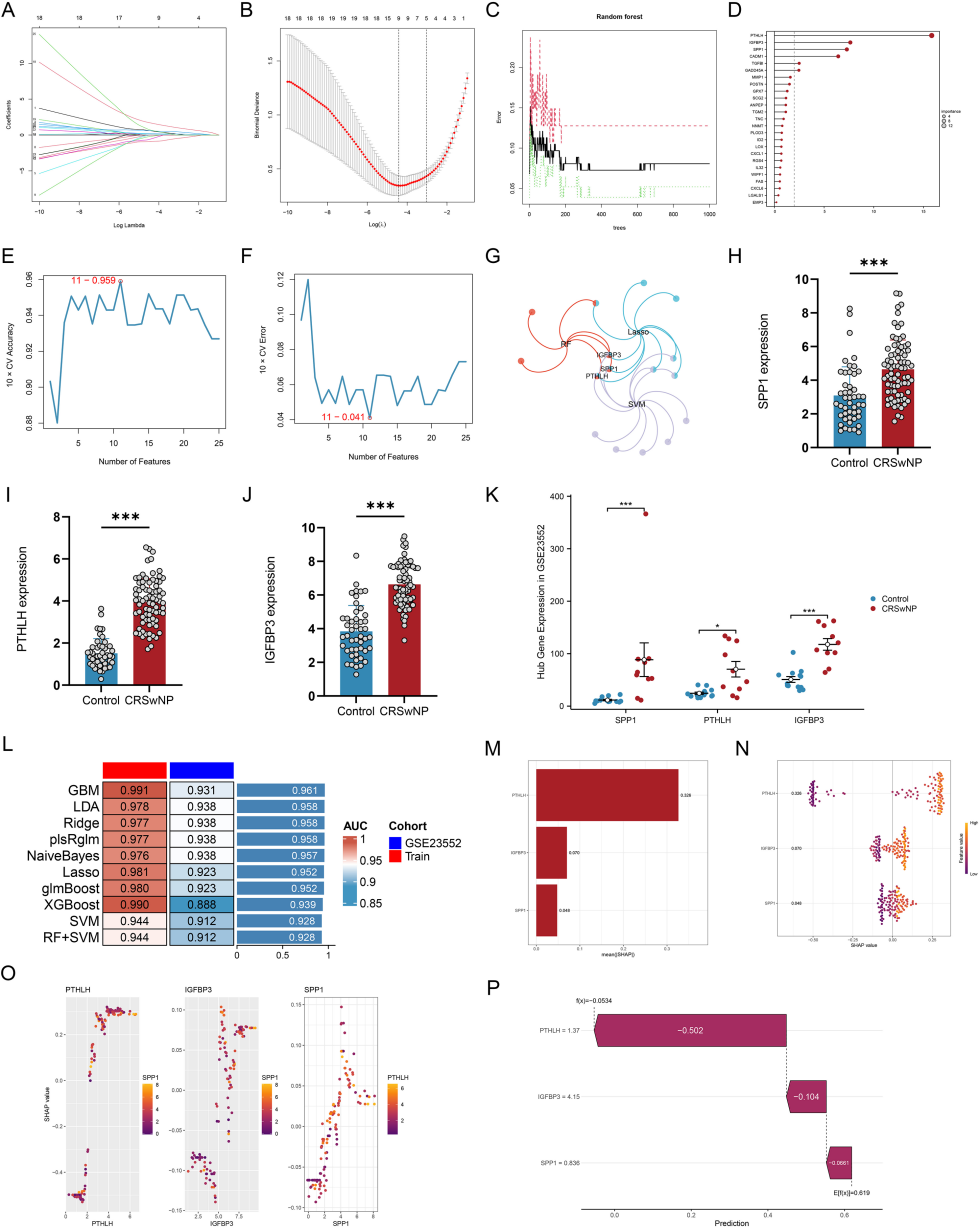
Machine-learning screening identifies SPP1, PTHLH, and IGFBP3 as EMT core genes with robust diagnostic performance. **(A, B)** LASSO coefficient profiles **(A)** and cross-validation curve for optimal penalty selection **(B)**. **(C, D)** Random forest training error **(C)** and variable importance ranking **(D)**. **(E, F)** SVM-RFE results showing cross-validated accuracy **(E)** and error **(F)** across different feature-set sizes. **(G)** Venn/overlap of features selected by LASSO, SVM-RFE, and random forest. **(H–J)** Expression of SPP1 **(H)**, PTHLH **(I)**, and IGFBP3 **(J)** in the training cohort. **(K)** External validation of core-gene expression in GSE23552. **(L)** AUC comparison of multiple classifiers in the training cohort and the external cohort. **(M–P)** SHAP interpretation, including mean absolute SHAP values **(M)**, SHAP summary plot **(N)**, dependence plots **(O)**, and an example waterfall plot **(P)**. *P < 0.05, **P < 0.01, ***P < 0.001. EMT, epithelial–mesenchymal transition; LASSO, least absolute shrinkage and selection operator; SVM-RFE, support vector machine–recursive feature elimination; SHAP, Shapley additive explanations; CRSwNP, chronic rhinosinusitis with nasal polyps; AUC, area under the curve.

Based on the 25 EMT candidate genes, multi-algorithm feature selection using LASSO, random forest, and SVM-RFE identified three EMT core genes by intersection: SPP1, PTHLH, and IGFBP3 (**Figures 3A–G**). In the training set, all three genes were significantly upregulated in CRSwNP tissues, and the same direction of differential expression was reproduced in the external validation dataset GSE23552 (**Figures 3H–K**). Multiple classification models were constructed using the three core genes; AUCs ranged from 0.944-0.991 in the training set and 0.888-0.938 in the external validation dataset (**Figure 3L**). SHAP analysis showed that all three genes contributed substantially to model prediction; PTHLH had the highest mean contribution, followed by IGFBP3 and SPP1. Overall, increased core-gene expression shifted model output toward the ‘CRSwNP’ category (**Figures 3M–P**).

### 3.4 Single-cell localization of core gene sources

**Figure 4:**
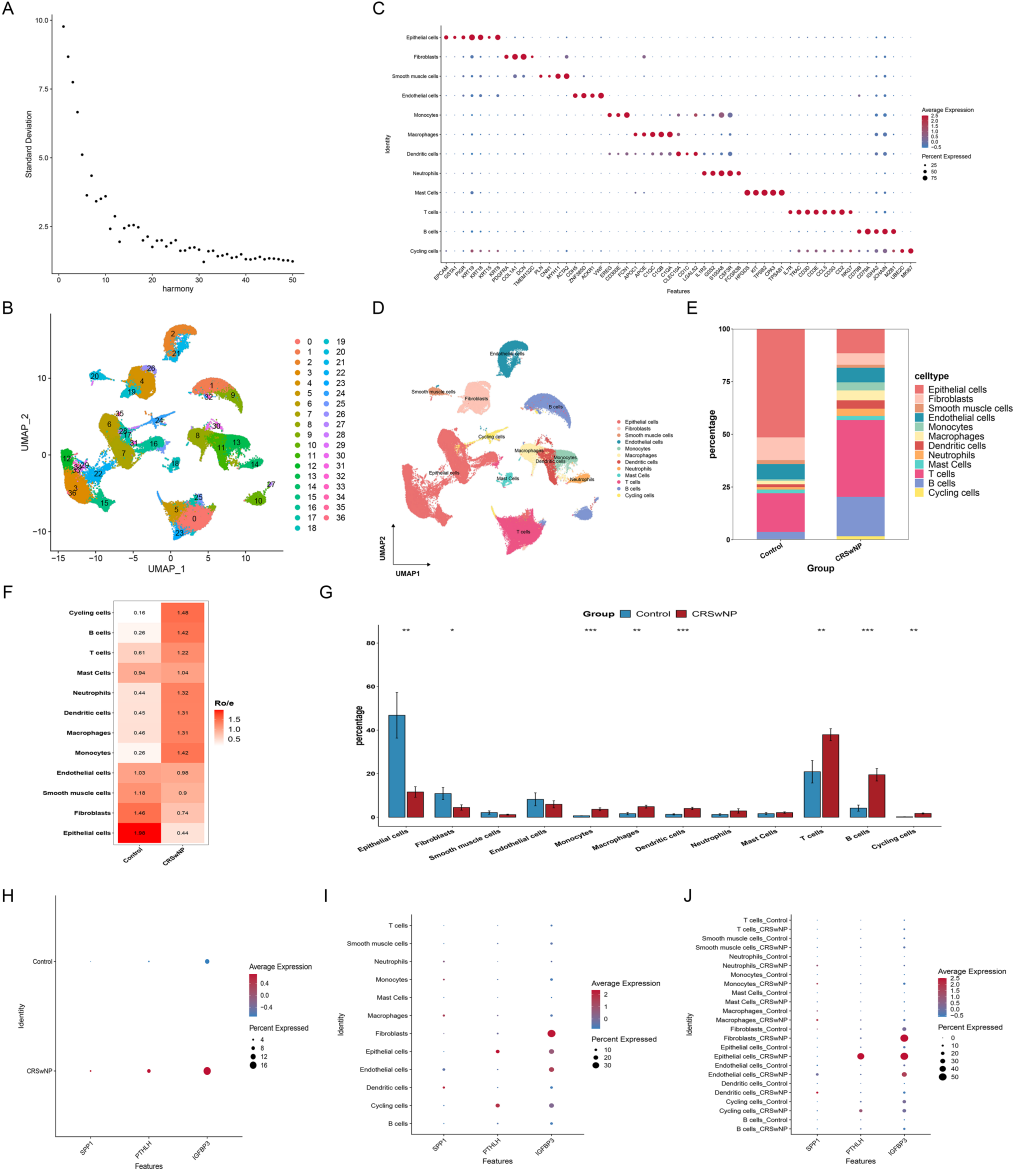
Single-cell transcriptomics maps EMT core genes to distinct cell types in CRSwNP. **(A)** Elbow plot used to select the number of Harmony-corrected PCA dimensions for downstream analyses. **(B)** UMAP visualization of clustered cells. **(C)** Dot plot of canonical markers for cell-type annotation (dot size, fraction of expressing cells; color, average expression). **(D)** UMAP of annotated major cell types. **(E)** Group-wise comparison of cellular composition. **(F)** Heatmap summarizing relative abundance changes across major cell types. **(G)** Bar plot of cell-type proportions (mean ± SD). **(H)** Dot plot comparing overall expression patterns of SPP1, PTHLH, and IGFBP3 between groups. **(I)** Cell-type–resolved expression of the three core genes across all annotated populations. **(J)** Cell-type–resolved expression stratified by group. *P < 0.05, **P < 0.01, ***P < 0.001. EMT, epithelial–mesenchymal transition; PCA, principal component analysis; UMAP, uniform manifold approximation and projection; CRSwNP, chronic rhinosinusitis with nasal polyps.

In the HRA000772 single-cell dataset, 100,082 cells were retained after stringent quality control and filtering, and 11 major cell populations were identified through unsupervised clustering followed by canonical marker-based annotation (**Figures 4A–D**; **Supplementary Figure S1**). Compared with controls, CRSwNP tissues showed relative enrichment of immune cell populations (e.g., monocytes/macrophages and dendritic cells), whereas the proportion of structural cells such as epithelial cells decreased (**Figures 4E–G**). Both the overall expression levels and the fraction of expressing cells for the three core genes were higher in CRSwNP than in controls (**Figure 4H**). Clear differences in cellular sources were observed: SPP1 was mainly expressed in myeloid cells, PTHLH was primarily enriched in epithelial cells, and IGFBP3 showed higher expression in fibroblasts and some structural cell subsets (**Figures 4I, J**).

### 3.5 Immune infiltration characteristics and pathway activities associated with the core genes

**Figure 5:**
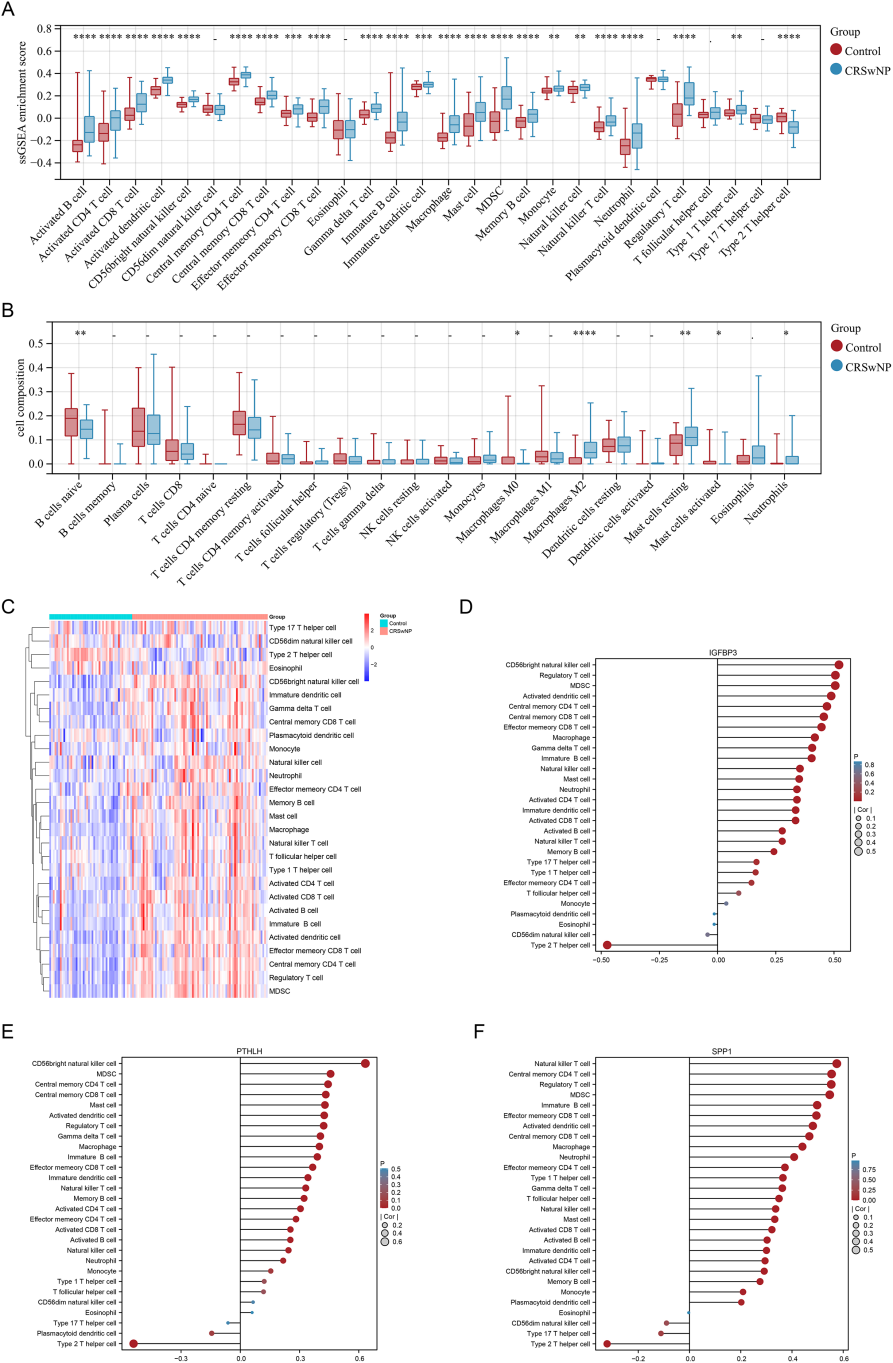
Immune infiltration landscape and its associations with EMT core genes in CRSwNP. **(A)** ssGSEA-derived immune cell enrichment scores in controls and CRSwNP. **(B)** Relative proportions of 22 immune cell subsets estimated by CIBERSORT. **(C)** Heatmap of ssGSEA immune scores across samples. **(D–F)** Lollipop plots summarizing correlations between immune infiltration scores and IGFBP3 **(D)**, PTHLH **(E)**, or SPP1 **(F)**. *P < 0.05, **P < 0.01, ***P < 0.001, ****P < 0.0001. EMT, epithelial–mesenchymal transition; CRSwNP, chronic rhinosinusitis with nasal polyps; ssGSEA, single-sample gene set enrichment analysis.

**Figure 6:**
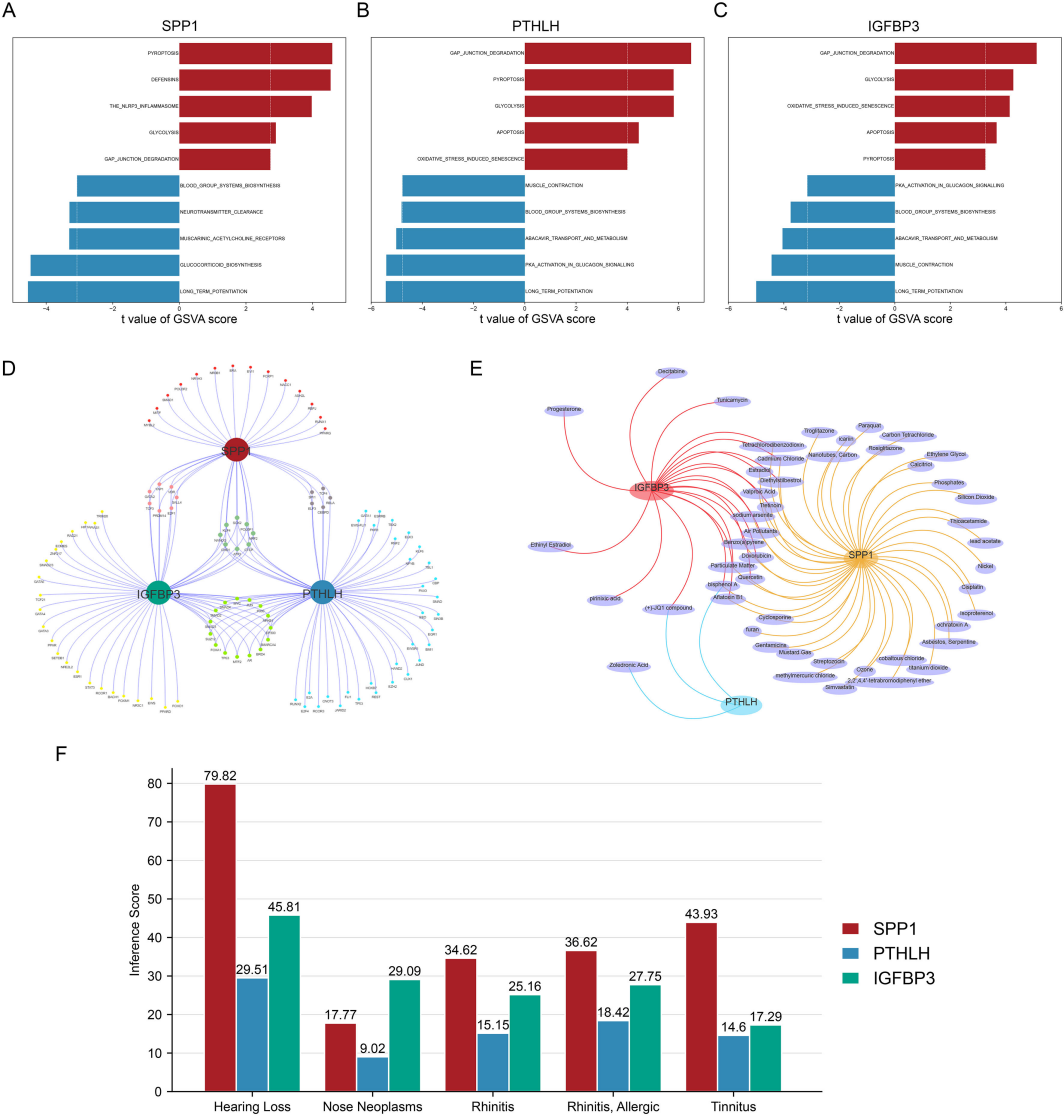
Pathway activity, regulatory network inference, and compound/disease associations of EMT core genes. **(A–C)** GSVA comparing pathway activity between high- and low-expression groups for SPP1 **(A)**, PTHLH **(B)**, and IGFBP3 **(C)**. **(D)** Predicted TF–gene regulatory network for SPP1, PTHLH, and IGFBP3 (miRNet/ChEA3). **(E)** CTD-derived chemical–gene interaction network, summarizing literature-curated compounds associated with the core genes. **(F)** CTD-based inference scores of disease associations for the core genes across selected otolaryngology-related conditions. EMT, epithelial–mesenchymal transition; GSVA, gene set variation analysis; TF, transcription factor; CTD, Comparative Toxicogenomics Database.

ssGSEA and CIBERSORT analyses suggested widespread alterations in immune infiltration in CRSwNP tissues, characterized by enhanced signals related to multiple immune cell types such as macrophages and mast cells, and reduced proportions of some resting/naive immune cells (e.g., naive B cells) (**Figures 5A–C**; **Supplementary Figure S2A**). Correlation analysis showed that SPP1, PTHLH, and IGFBP3 were significantly associated with infiltration scores of multiple immune cell types, and exhibited relatively consistent positive correlations with macrophages, mast cells, and myeloid-derived suppressor cells (MDSCs) (**Figures 5D–F**; **Supplementary Figure S2B**). At the pathway level, GSVA analysis stratified by core-gene expression showed that the high-expression group tended to activate cell death/inflammation-related processes (e.g., pyroptosis and inflammasome-related signaling), glycolysis, and oxidative stress pathways, whereas pathways related to neurotransmitter clearance and hormone metabolism showed a suppressive trend (**Figures 6A–C**).

### 3.6 Upstream regulatory networks, compound interactions, and disease associations

A TF-gene network was constructed using ChEA3 via miRNet (**Figure 6D**). SPP1, PTHLH, and IGFBP3 corresponded to 34, 60, and 53 candidate TFs, respectively, and 8 TFs were predicted to regulate all three core genes. CTD analysis indicated that 52, 3, and 27 compounds were associated with SPP1, PTHLH, and IGFBP3, respectively; notably, bisphenol A showed literature-level associations with all three core genes (**Figure 6E**). In addition, all three core genes showed associations with otorhinolaryngology-related disease terms such as rhinitis/allergic rhinitis (**Figure 6F**).

### 3.7 Dual-center qRT-PCR validation and histological evidence

**Figure 7:**
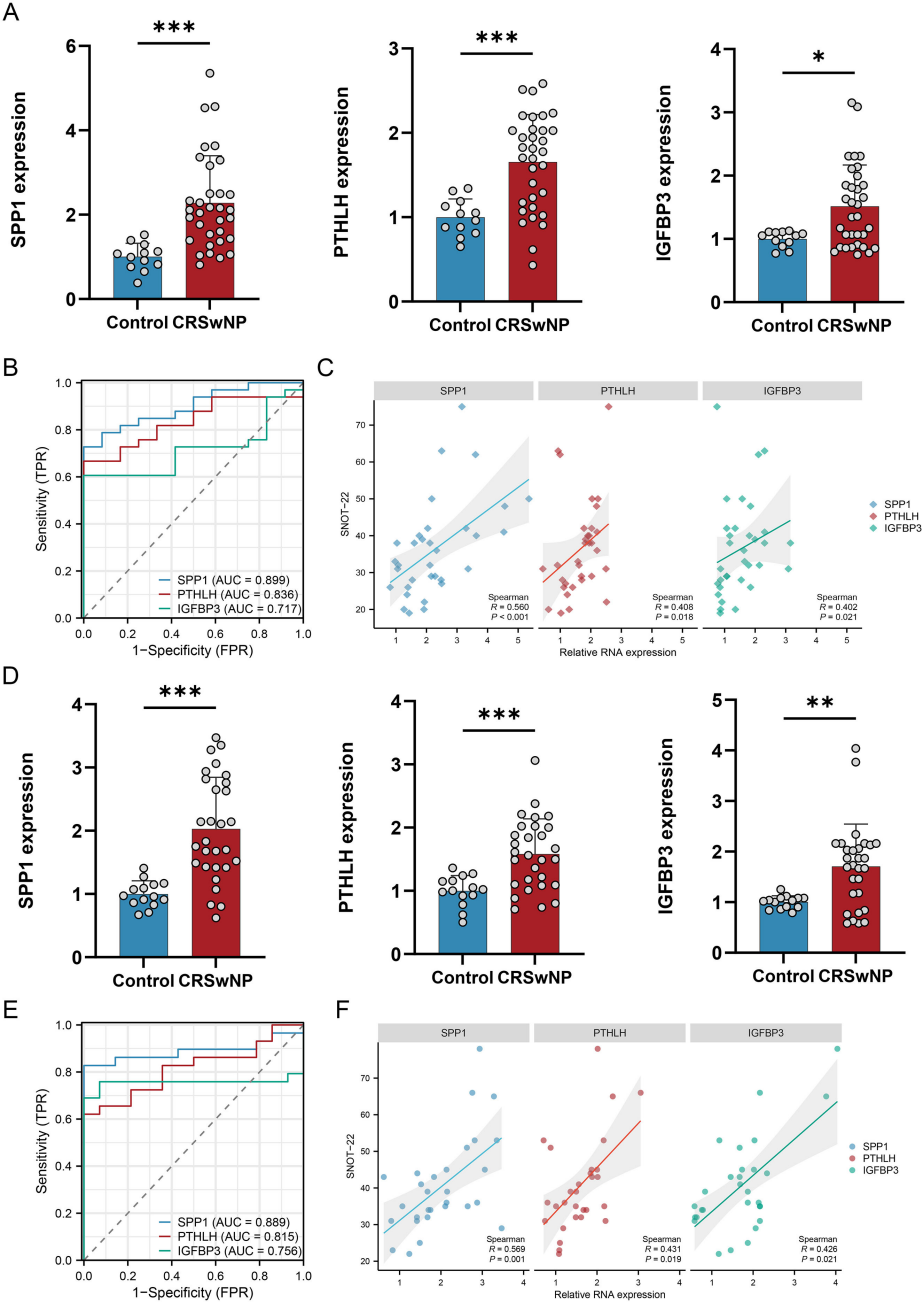
Dual-center experimental validation and clinical relevance of EMT core genes. **(A)** qRT-PCR validation of SPP1, PTHLH, and IGFBP3 in the CQ cohort. **(B)** ROC curves assessing the diagnostic performance of each gene in the CQ cohort. **(C)** Spearman correlations between gene expression and symptom burden (SNOT-22) in the CQ cohort. **(D)** qRT-PCR validation in the independent LN cohort. **(E)** ROC curves for the LN cohort. **(F)** Spearman correlations between gene expression and SNOT-22 in the LN cohort. *P < 0.05, **P < 0.01, ***P < 0.001. EMT, epithelial–mesenchymal transition; qRT-PCR, quantitative real-time PCR; ROC, receiver operating characteristic; SNOT-22, 22-item Sino-Nasal Outcome Test; CQ, Chongqing cohort; LN, Liaoning cohort; CRSwNP, chronic rhinosinusitis with nasal polyps.

**Figure 8:**
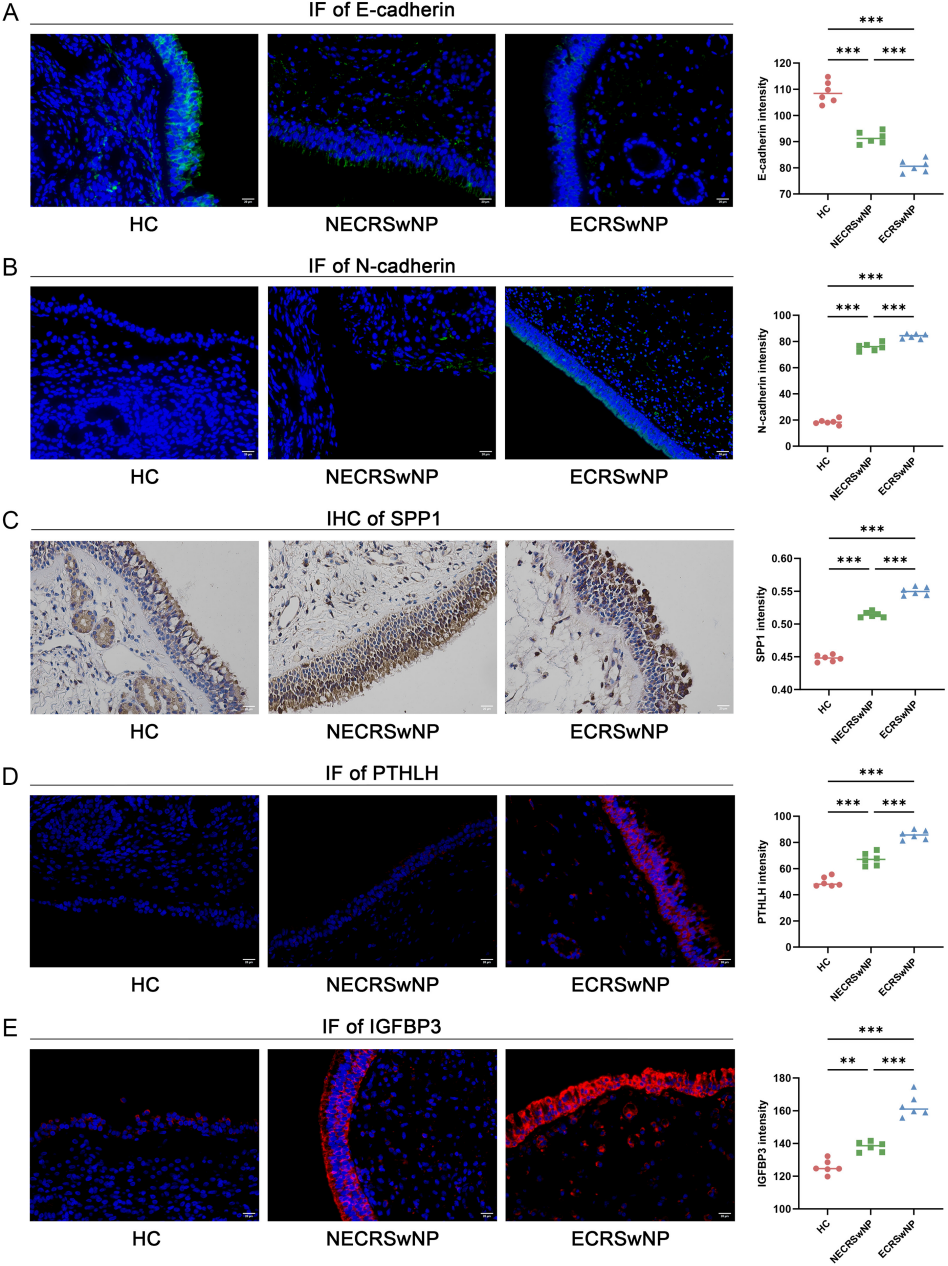
Histological validation of EMT markers and EMT core genes across CRSwNP endotypes. **(A)** IF staining of E-cadherin in healthy controls (HC), non-eosinophilic CRSwNP (NECRSwNP), and eosinophilic CRSwNP (ECRSwNP), with quantitative fluorescence-intensity analysis. **(B)** IF staining and quantification of N-cadherin. **(C)** IHC staining of SPP1 with semi-quantitative/quantitative assessment of staining intensity. **(D)** IF staining and quantification of PTHLH. **(E)** IF staining and quantification of IGFBP3. Nuclei were counterstained with DAPI (blue). **P < 0.01, ***P < 0.001. EMT, epithelial–mesenchymal transition; CRSwNP, chronic rhinosinusitis with nasal polyps; HC, healthy control; NECRSwNP, non-eosinophilic CRSwNP; ECRSwNP, eosinophilic CRSwNP; IF, immunofluorescence; IHC, immunohistochemistry.

In two independent clinical cohorts (CQ and LN), qRT-PCR consistently showed significant upregulation of SPP1, PTHLH, and IGFBP3 in CRSwNP (**Figures 7A, D**). ROC analysis showed that the AUCs of the three core genes in the CQ cohort were 0.899, 0.836, and 0.717, respectively, and the corresponding AUCs in the LN cohort were 0.889, 0.815, and 0.756 (**Figures 7B, E**). Core-gene expression correlated positively with SNOT-22 scores, with SPP1 showing the most consistent association with symptom burden (CQ: r = 0.560, P < 0.001; LN: r = 0.569, P = 0.001) (**Figures 7C, F**). In histological validation using LN samples, E-cadherin showed a decreasing trend across HC, NECRSwNP, and ECRSwNP, whereas N-cadherin showed an increasing trend, supporting EMT activation. Moreover, SPP1 (IHC), PTHLH(IF), and IGFBP3 (IF) showed stronger signals in ECRSwNP (**Figures 8A–E**).

## 4 Discussion

In CRSwNP, persistent inflammation, epithelial barrier dysfunction, and mucosal remodeling often drive each other and form a positive feedback loop. EMT is considered one of the key histological and molecular events linking ‘barrier disruption-remodeling aggravation-inflammation maintenance’. EMT may weaken inter-epithelial junctions, promote reconstruction of matrix components, and enhance migratory/invasive-like phenotypes, thereby facilitating polyp formation and aberrant mucosal architecture (1, 18, 49).

To address the unmet need for robust EMT-associated molecular features in CRSwNP, we integrated internal RNA-seq data with multiple public datasets to construct a training set. After batch effect correction, candidate genes were obtained via combined differential expression analysis, key WGCNA modules, and intersection with an EMT gene set. Multi-algorithm intersection using LASSO, SVM-RFE, and random forest further identified three core genes—SPP1, PTHLH, and IGFBP3—forming a minimal, cross-platform EMT signature that was consistently reproduced across cohorts. In this study, “core genes” are defined by cross-cohort reproducibility and statistical robustness supported by a multi-layer evidence chain, rather than by systematic ranking of EMT markers according to clinical associations. Single-cell transcriptomics was then used to localize the major source cell types of the core genes at cellular resolution, while immune infiltration and pathway activity analyses provided complementary, hypothesis-generating biological context. Finally, consistent cross-platform and cross-region validation was achieved through dual-center clinical samples (qRT-PCR) and histological assays (IHC/IF), supporting a multi-layered evidence framework from population-level association to source-cell localization and ultimately to histological corroboration. Direct comparative evaluation against established EMT markers across clinical dimensions such as severity, endotype, or recurrence will require outcome-oriented prospective cohorts.

EMT typically occurs within a complex inflammatory microenvironment, and its initiation and maintenance can be influenced by multiple factors, including immune cell infiltration lineages, cytokine networks, hypoxia, and metabolic reprogramming (18, 49). Our immune infiltration analyses suggested enhanced signals for multiple immune cell types, and all three core genes showed significant correlations with diverse immune infiltration scores, supporting their potential involvement in an amplifying loop of immune-structural cell interactions. The single-cell atlas further showed concomitant enrichment of immune cells and altered states of structural cells in CRSwNP tissues, with the core genes displaying distinct source cell distributions, consistent with the framework of inflammatory heterogeneity and cell-state remodeling described in previous CRS single-cell studies (45). At the pathway level, the high core-gene expression group tended to be enriched for inflammasome/pyroptosis-related signaling, glycolysis, oxidative stress, and cellular senescence/apoptosis. Recent studies of nasal mucosal inflammation have increasingly emphasized the role of the inflammasome and pyroptosis in disrupting epithelial homeostasis. For example, in eosinophilic CRS, IL-21 has been reported to induce pyroptosis of Treg cells via the Akt-mTOR-NLRP3-caspase-1 axis and thereby affect immune homeostasis (50). In CRS/nasal polyp-related research, a hypoxia-HIF-1α-NLRP3 axis has been proposed to influence epithelial differentiation programs (51), and pyroptosis activation has also been suggested to disturb differentiation of nasal epithelial basal cells and bias them toward goblet cell differentiation, aggravating epithelial abnormalities and a mucous phenotype (52). These lines of evidence align with our enrichment results, suggesting that the core genes may reflect a tissue state characterized by both high inflammatory load and high remodeling activity, rather than serving merely as single inflammatory markers.

Among the three core genes, SPP1 was consistently upregulated in the training set, external validation cohort, and dual-center clinical samples, and showed a relatively strong positive correlation with nasal inflammatory cell signals and SNOT-22 symptom scores, indicating notable clinical relevance. Previous studies have reported elevated SPP1 expression in CRSwNP tissues and its association with local inflammatory regulation (53). More direct mechanistic evidence suggests that, under eosinophilic inflammatory conditions, eosinophil-derived SPP1 can stimulate nasal fibroblasts, promote inflammation and tissue remodeling, and correlate with disease severity (54). From a molecular function perspective, SPP1, as a secreted matrix/cytokine-like molecule, can interact with receptors such as CD44 via ligand-receptor binding and participate in cell adhesion, migration, and recruitment of inflammatory cells, and can also cooperate with integrins to mediate multiple inflammation-fibrosis processes (55). Our single-cell results indicated that SPP1 is mainly expressed by myeloid cell populations such as macrophages, providing cellular support for the hypothesis that immune cell-derived SPP1 may influence epithelial/stromal cell states through paracrine signaling and promote ECM remodeling and EMT maintenance. Together with clinical evidence suggesting that nasal secretion SPP1 may help distinguish CRSwNP endotypes and predict severity, SPP1 may represent a candidate biomarker and intervention target with both stratification and translational potential (56). Blocking the interaction between SPP1 and its receptors or reducing its expression may not only alleviate inflammatory cell infiltration within polyp tissues but also suppress excessive fibrotic remodeling. Anti-SPP1 antibody interventions have been explored in murine asthma models, where airway inflammation and remodeling markers were reportedly reduced, suggesting that similar strategies may warrant exploration in nasal polyposis.

PTHLH (encoding PTHrP) was robustly upregulated in this study and was primarily localized to epithelial cell populations in single-cell analyses; histological staining also suggested increased expression in diseased tissues. This is consistent with its biological feature as an epithelial-derived paracrine factor involved in epithelial-stromal interactions. Although direct mechanistic evidence for PTHLH in CRSwNP remains limited, prior transcriptomic studies of sinus mucosa in CRS reported that PTHLH was among the most significantly upregulated glandular-related genes, with higher mRNA and protein levels than normal mucosa, corresponding to marked submucosal gland hyperplasia in CRS patients (57). Outside the CRS field, PTHrP has been reported to promote EMT-like programs in other systems. For example, in prostate cancer, Ongkeko et al. reported that PTHrP overexpression promoted acquisition of mesenchymal phenotypes by epithelial cells and enhanced cancer cell invasion and metastasis; conversely, PTHrP knockdown reversed EMT marker changes and suppressed invasiveness (58). Similarly, in a renal fibrosis model, PTHrP was shown to synergize with TGF-β1 to induce tubular epithelial EMT via activation of ERK signaling (59). Together, these findings support PTHLH as an EMT-associated candidate gene in CRSwNP and motivate future cell-type-specific functional perturbation and pathway interrogation in relevant airway epithelial and stromal models (60). Importantly, our data are cross-sectional and associative, and do not establish a causal role for PTHLH (or its receptor signaling axis) in polyp development.

IGFBP3 is one of the most abundant insulin-like growth factor (IGF) binding proteins in plasma and regulates IGF half-life, bioavailability, and tissue accessibility, thereby influencing processes such as cell growth and differentiation (61). In tissue remodeling, IGFBP3 is more commonly linked to pro-fibrotic activity. In idiopathic pulmonary fibrosis (IPF), IGFBP3 (and IGFBP5) is increased in lung tissue and in fibroblasts and can induce normal lung fibroblasts to produce ECM components such as collagen I and fibronectin, supporting its involvement in ECM deposition and fibrosis progression (62). In addition, IGFBP3 can interact with other growth factors, for example by enhancing VEGF-driven angiogenic signaling, which may contribute to the rich neovascularization in nasal polyps and facilitate inflammatory exudation (63). Moreover, beyond acting as a stromal-derived paracrine factor, IGFBP3 can be induced in epithelial cells by stimuli such as TGF-β and can amplify TGF-β/SMAD signaling in an IGF-independent manner, upregulating key EMT transcription factors and thereby enhancing epithelial migration/invasion and EMT-like phenotypes (64). In addition, an IGFBP3/TMEM219 axis involved in cell death and homeostatic regulation has been proposed and validated in other tissue systems (65), suggesting additional IGF-independent pathways through which IGFBP3 may influence tissue homeostasis and remodeling.

In addition to expression- and pathway-level evidence, our database-based association analyses provided testable hypotheses for mechanism-oriented studies. TF prediction based on miRNet-ChEA3 suggested that multiple upstream factors related to inflammatory stress and chromatin regulation may jointly participate in transcriptional regulation of the core genes. It should be emphasized that these TF results are inferences derived from databases and algorithms, and their biological validity requires experimental verification, such as ChIP-qPCR/ChIP-seq. Meanwhile, CTD suggested literature-level associations between the core genes and multiple otorhinolaryngology-related diseases and chemical exposures, providing clues for drug repurposing or environment-gene investigations; however, these associations do not imply causality and require targeted validation in CRSwNP models.

The major strengths of this study are as follows. First, by integrating multiple cohorts and applying multi-algorithm feature selection, we obtained a stable core gene signature across platforms and achieved consistent validation in an external dataset and dual-center clinical samples, reducing the risk of cohort-specific findings. Second, by combining population-level associations from bulk transcriptomics with source cell localization at single-cell resolution, we established a relatively closed explanatory framework linking ‘candidate biomarkers-source cells-potential interaction networks. Third, by incorporating histological evidence and clinically relevant phenotypes (e.g., eosinophilic endotype and correlation with SNOT-22), we enhanced the clinical interpretability of the findings and generated clinically grounded, testable hypotheses for future stratification and translational studies.

Although this study is exploratory and does not support causal inference or an immediately deployable stratification algorithm, it motivates a clear and testable translational hypothesis: a parsimonious EMT-associated three-gene signature (SPP1-PTHLH-IGFBP3) may reflect remodeling/EMT activity in CRSwNP and be relevant to persistence, postoperative recurrence, and therapeutic response. Future prospective longitudinal cohorts with prespecified sampling and clinically meaningful endpoints (e.g., endoscopic polyp score and symptom burden [SNOT-22], time-to-recurrence/revision, and response to biologics where applicable) should assess whether this signature provides incremental predictive value beyond EPOS-guided clinical assessment and inflammatory endotyping. This evaluation could address three clinically actionable questions: whether the signature stratifies recurrence risk after ESS; whether it indicates a remodeling/EMT-predominant tissue state despite comparable inflammatory burden; and whether it is associated with differential response trajectories under standard therapy or biologics. In our histological validation, stronger signals in the eosinophilic endotype suggest that this signature may be most informative when interpreted alongside EPOS-defined type 2 disease and current biologic decision pathways, as a remodeling-focused complement rather than a replacement for inflammatory endotyping. Translation will require standardized assays with prespecified thresholds (analytical validity), reproducible outcome associations with independent validation and demonstrated incremental predictive contribution (clinical validity), and ultimately prospective evidence that biomarker-informed decision-making improves clinically meaningful outcomes (clinical utility).

This study also has limitations. First, although multi-cohort integration and batch-effect correction were applied, residual biases due to platform and population differences may remain. As a result, genes that are significant only in specific datasets could have been missed, and attention might be biased toward universally present but moderately sized effects. Second, the analyses were primarily based on cross-sectional data and thus cannot dynamically evaluate changes in core-gene expression with treatment or disease course, nor can they assess predictive value for recurrence. Future work should evaluate core-gene expression in large prospective nasal polyp cohorts with standardized follow-up (e.g., postoperative recurrence and treatment response, including biologics) to test prognostic/predictive value. Third, mechanistic causality cannot be established without functional perturbation. Although multi-omics analyses and clinical sample validation support the relevance of the core genes, further cell-type-specific perturbation studies in relevant cellular models and *in vivo* systems are required. In ongoing work, we are preparing to establish a macrophage-specific conditional Spp1 knockout mouse model to interrogate the contribution of myeloid-derived SPP1 to EMT-associated remodeling phenotypes in CRSwNP. Additionally, control tissues were derived from septal deviation patients, as obtaining truly healthy sinonasal mucosa from volunteers is generally not ethically feasible; although CRS was excluded by clinical assessment and endoscopy/radiology, subtle baseline inflammatory bias cannot be completely ruled out.

## 5 Conclusions

Through multi-cohort transcriptomic integration, network analysis, and multi-algorithm machine learning, SPP1, PTHLH, and IGFBP3 were identified as EMT-related core genes in CRSwNP and were validated using an external dataset, single-cell transcriptomic analysis, and dual-center qRT-PCR and histological assays. These genes were consistently associated with signatures of immune microenvironment alteration and mucosal remodeling, providing a robust, hypothesis-generating molecular framework that links population-level signals to cellular sources. Importantly, the present findings are not yet clinically actionable for patient stratification or therapeutic selection, as outcome-based associations (e.g., treatment response and postoperative recurrence) were not evaluated. Future studies should prioritize longitudinal and prospective cohorts with clinically meaningful endpoints, together with *in vivo* mechanistic experiments, to clarify causality and assess translational potential.

## Data Availability

The raw data supporting the conclusions of this article will be made available by the authors, without undue reservation.
