# Supplementary material for: Identification and dual-center histological validation of EMT core genes in chronic rhinosinusitis with nasal polyps: an integrated multi-cohort transcriptomic and single-cell analysis
Source: Front Immunol. 2026 Mar 17;17:1774236. doi: 10.3389/fimmu.2026.1774236 (PMC13035518; doi:10.3389/fimmu.2026.1774236)
Supplement: Supplementary file 1 [file DataSheet1.pdf]

Supplementary Material

1 Supplementary Tables

1.1 Supplementary Table S1. Summary of patient characteristics and methodology used in the Chongqing cohort

Variable	Control	CRSwNP	P-value
Total of patient,n	17	40	
Age,yr mean±SD	46.29(15.72)	43.98(13.60)	0.350
Male,n(%)	10(58.82)	22(55.00)	0.790
BMI,kg/m²mean±SD	23.68(2.09)	24.19(3.12)	0.540
Smoker,n(%)	4(23.52)	13(32.50)	0.498
SNOT-22 score, Median (IQR)	-	32.5 (26.0–42.0)	
Endotype status	-	24 eosinophilic, 16 non-eosinophilic	
Methodology used			
mRNA sequencing	5	7	
Age(mean±SD)	37.00(16.98)	35.14(13.58)	0.850
Male,n(%)	2(40.00)	2(28.57)	0.999
BMI,kg/m²mean±SD	22.69(1.82)	22.72(2.78)	0.992
Smoker,n(%)	0(0.00)	1(14.29)	0.999
SNOT-22 score, Median (IQR)	-	25.0 (17.0–49.5)	
Endotype status	-	3 eosinophilic, 4 non-eosinophilic	
qRT-PCR	12	33	
Age(mean±SD)	50.17(13.14)	45.85(13.05)	0.330
Male,n(%)	8(66.67)	20(60.61)	0.999
BMI,kg/m²mean±SD	24.09(2.12)	24.50(3.14)	0.680
Smoker,n(%)	4(33.33)	12(36.36)	0.999
SNOT-22 score, Median (IQR)	-	33.0 (28.0–41.0)	

Endotype status	-	21 eosinophilic,<br>12 non-eosinophilic
Correlation analysis		33
IHC/IF	6	12

BMI, Body Mass Index; SNOT-22, Sino-Nasal Outcome Test-22; qRT-PCR, Quantitative real-time; IHC, immunohistochemistry; IF, immunofluorescence

1.2 Supplementary Table S2. Summary of patient characteristics and methodology used in the Liaoning cohort

Variable	Control	CRSwNP	P-value
Total of patient,n	14	29	
Age,yr mean±SD	43.46(14.39)	45.31(13.58)	0.690
Male,n(%)	8(57.14)	17(58.62)	0.859
BMI,kg/m²mean±SD	23.26(2.47)	24.12(2.82)	0.350
Smoker,n(%)	4(28.57)	7(24.14)	0.713
SNOT-22 score, Median (IQR)	-	36.0 (32.0–45.0)	
Methodology used			
qRT-PCR	14	29	
Correlation analysis		29	

BMI, Body Mass Index; SNOT-22, Sino-Nasal Outcome Test-22

1.3 Supplementary Table S3. Summary of transcriptomic datasets used in this study

Dataset	Data type	CRSwNP	Control	Role in study
GSE72713	Bulk RNA-seq	6 nasal polyp tissues from CRSwNP	3 Sphenoid sinus mucosa from the healthy control	Training (integrated)
GSE136825	Bulk RNA-seq	42 nasal polyp tissues from CRSwNP	28 inferior turbinates from the healthy control	Training (integrated)
GSE179265	Bulk RNA-seq	17 nasal polyp tissues from CRSwNP	7 uncinate process from the healthy control	Training (integrated)
GSE198950	Bulk RNA-seq	5 Ethmoidal tissues from CRSwNP	4 inferior turbinates from the healthy control	Training (integrated)
CQ cohort	Bulk RNA-seq	7 nasal polyp tissues from CRSwNP	5 uncinate process from the healthy control	Training (integrated)
GSE23552	Microarray	10 nasal polyp tissues from CRSwNP	13 normal nasosinus tissue from the healthy control	External validation
HRA000772	scRNA-seq	11 nasal polyp tissues from CRSwNP	5 Ethmoid/sphenoid sinus mucosa from healthy controls	Cellular source mapping

1.4 Supplementary Table S4. The list of primer sequences

Gene	Forward primer (5′ → 3′)	Reverse primer (5′ → 3′)
GAPDH	CACCCACTCCTCCACCTTTGAC	GTCCACCACCCTGTTGCTGTAG
SPP1	AGCAGAATCTCCTAGCCCCA	CTGGCTGTCCACATGGTCAT
PTHLH	TCGCAGAAATCCACACAGCT	ACGGGGTGGTTCTTTGTGTT
IGFBP3	GCATGCTAAAGACAGCCAGC	GGAATGTGTACACCCCTGGG

1.5 Supplementary Table S5. The list of Antibody.

Antibody	Cat No.	Vendor	Application
E-cadherin	20874-1-AP	proteintech	IF,1:400
N-cadherin	22018-1-AP	proteintech	IF,1:400
SPP1	30200-1-AP	proteintech	IHC,1:400
PTHLH	PA5-57493	Invitrogen	IF,1:400
IGFBP3	10189-2-AP	proteintech	IF,1:400

2.Supplementary Figures

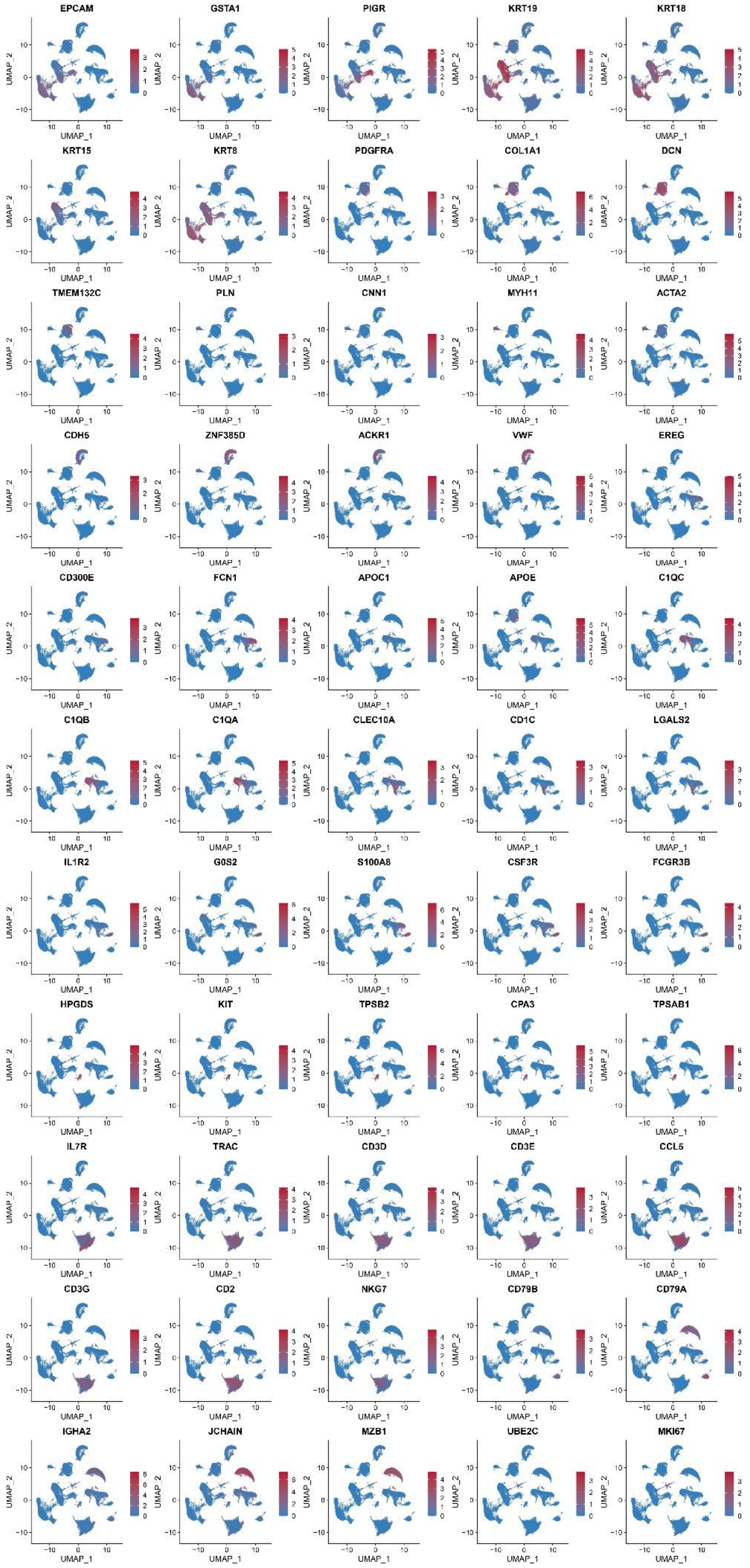
EPCAM
GSTA1
PIGR
KRT19
KRT18
KRT15
KRT8
PDGFRA
COL1A1
DCN
TMEM132C
PLN
CNN1
MYH11
ACTA2
CDH5
ZNF385D
ACKR1
VWF
EREG
CD300E
FCN1
APOC1
APOE
C1QC
C1QB
C1QA
CLEC10A
CD1C
LGALS2
IL1R2
G0S2
S100A8
CSF3R
FCGR3B
HPGDS
KIT
TPSB2
CPA3
TPSAB1
IL7R
TRAC
CD3D
CD3E
CCL5
CD3G
CD2
NKG7
CD79B
CD79A
IGHA2
JCHAIN
MZB1
UBE2C
MKI67

Supplementary Figure S1. Canonical marker gene expression for cell-type annotation in the scRNA-seq atlas. Feature plots show the normalized expression of representative lineage markers projected onto the UMAP embedding (each dot represents a single cell; color scale from low [blue] to high [red]). Markers include epithelial cells (EPCAM, GSTA1, PIGR, KRT19, KRT18, KRT15, KRT8), fibroblasts (PDGFRA, COL1A1, DCN), smooth muscle/pericyte-like stromal cells (TMEM132C, PLN, CNN1, MYH11, ACTA2), endothelial/vascular cells (CDH5, ZNF385D, ACKR1, VWF, EREG), myeloid subsets including monocytes/macrophages/dendritic cells (CD300E, FCN1, APOC1, APOE, C1QA, C1QB, C1QC, CLEC10A, CD1C, LGALS2) and neutrophil-associated markers (IL1R2, G0S2, S100A8, CSF3R, FCGR3B), mast cells (HPGDS, KIT, TPSB2, CPA3, TPSAB1), T cells/cytotoxic T cells (IL7R, TRAC, CD3D, CD3E, CCL5), NK/cytotoxic lymphocytes (NKG7), B cells (CD79A, CD79B), plasma cells (IGHA2, JCHAIN, MZB1), and cycling cells (UBE2C, MKI67).

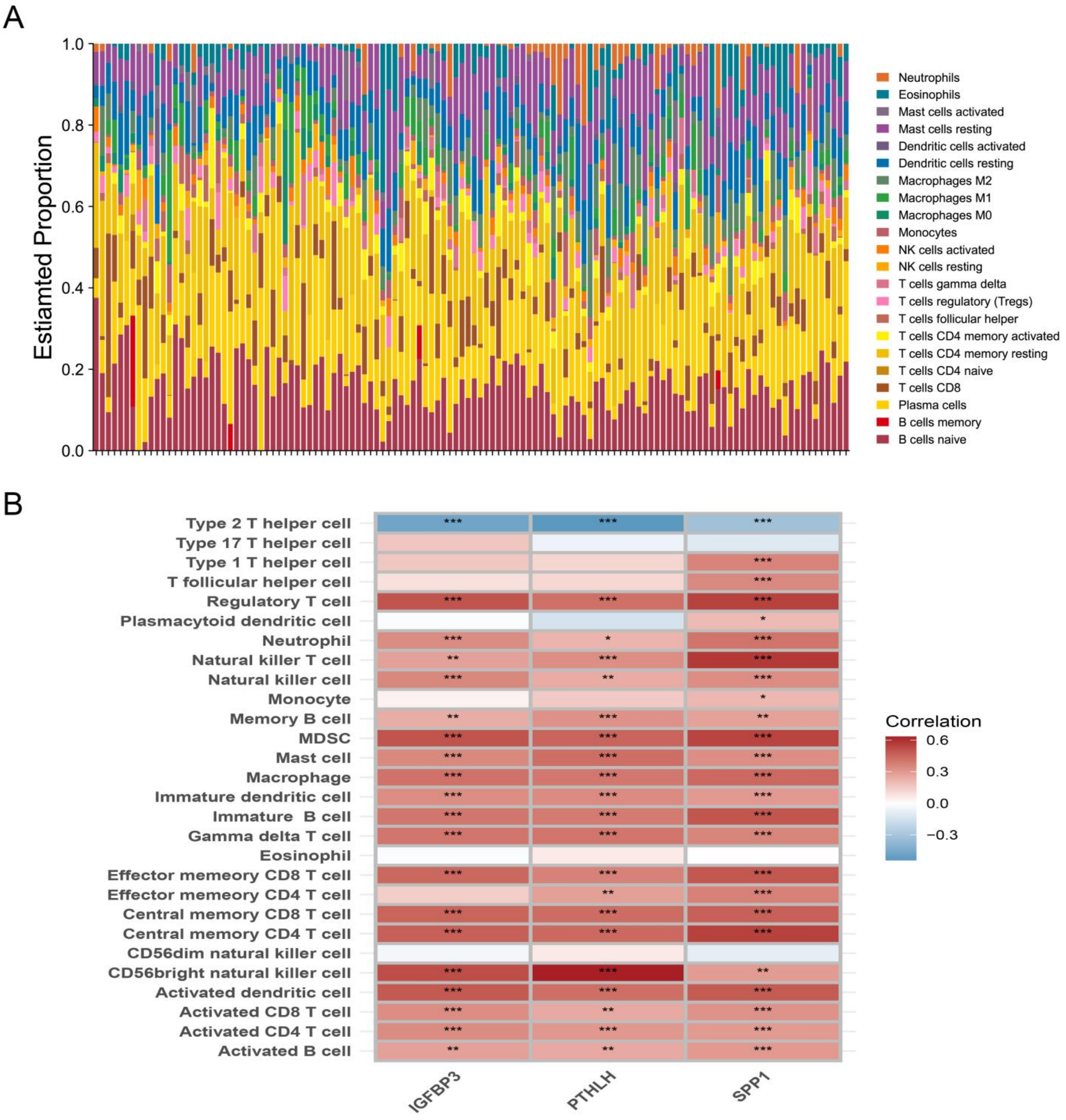

Supplementary Figure S2. Sample-level immune composition and gene–immune association details for immune infiltration analyses.(A) Stacked bar plot showing CIBERSORT-inferred relative composition of 22 immune cell subsets for each sample. Each bar represents one sample and sums to 100%. (B) Spearman correlation heatmap between the expression of SPP1/PTHLH/IGFBP3 and ssGSEA-derived immune cell enrichment scores across samples. Color indicates correlation coefficient (ρ), with positive and negative correlations shown on a continuous scale; significance is denoted by asterisks (*P < 0.05, **P < 0.01, ***P < 0.001, ****P < 0.0001), unless otherwise specified. ssGSEA, single-sample gene set enrichment analysis.
